# Highlight: Yellow is the New Black for Gobi Desert Lizards

**DOI:** 10.1093/gbe/evac095

**Published:** 2022-07-07

**Authors:** Casey McGrath

An animal’s skin, fur, or feather colors can either be used to attract attention or avoid it, allowing individuals to entice a mate or to escape detection by predators. Pigmentation may also affect physiological features such as thermoregulation and susceptibility to UV damage. All of these represent potential selective pressures that could drive changes in pigmentation or coloration between species or among populations. The variegated toad-headed lizard *Phrynocephalus versicolor* lives amid the colorful sands of the Gobi Desert. As its name in Latin suggests, populations of this lizard exhibit color differences across the species’ geographic range. In a new study published in *Genome Biology and Evolution*, an international team of collaborators led by corresponding authors Yuanting Jin from China Jiliang University and Diana Aguilar Gómez from the University of California Berkeley sought to reveal the genetic changes and selection pressures giving rise to variation in this colorful lizard (Jin et al. 2022).

The sands of the Gobi Desert range from black to light yellow, providing differently colored substrates for *P. versicolor.* A previous study by Aguilar Gómez, Jin, and Tong showed that the lizards’ skin color tended to match the color of the sand in its environment (Tong et al. 2019), and that this was genetically determined: lizards transferred to a different locality did not change color. These suggested differences among populations in the production and storage of melanin in melanophores (producing black/brown), as well as carotenoid pigments in xanthophores (producing yellow).

**Fig. 1.**
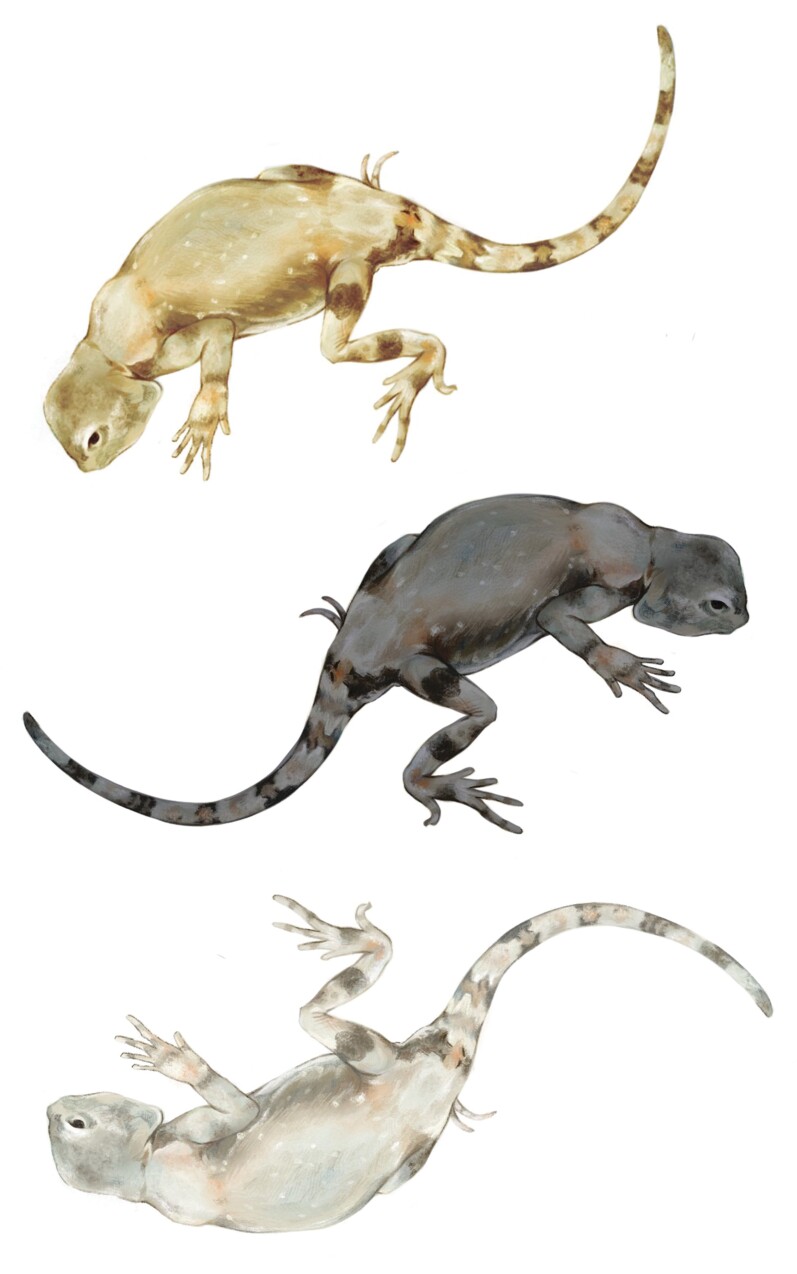
Three color morphs of *Phrynocephalus versicolor:* a yellow morph from Ejin Banner (top), a black morph from Heishankou (middle), and a pale yellow morph from Guazhou County (bottom). Illustration by Philippa Steinberg.

To understand the genetic basis of this intriguing color polymorphism, the authors performed microscopic analyses of skin samples and generated the first genome assembly for *P. versicolor*. They further sequenced the genomes of 94 individuals from three populations, each exhibiting a different skin color: lizards from Ejin Banner were yellow, those from Guazhou County were a paler shade of yellow, and those from Heishankou were black (fig. 1). Genetic analyses revealed that the pale yellow and black lizards from Guazhou County and Heishankou were more closely related to each other than either was to the yellow lizards of Ejin Banner.

The authors then assessed the genomes for evidence of positive selection and performed transcriptomic analyses to identify genes that were differentially expressed between populations. This resulted in the identification of several candidate genes that had been previously reported to be involved in pigmentation. One expected gene, however, was notably absent from this list. According to Aguilar Gómez, “The most surprising finding was that among the candidates, we did not find *mc1r*, which is the gene that has been consistently found in melanic vs. nonmelanic study systems,” including both reptiles and mammals.

Instead, the authors identified *slc2a11* and *akap12* as the strongest candidates for genes underlying color variation in *P. versicolor*. *Akap12* was the most divergent gene in the melanic (black) population compared with the nonmelanic (yellow) populations, and a single-nucleotide polymorphism in this gene was associated with reflectance. In contrast, *slc2a11* was more highly expressed in the yellow Guazhou County population than in the black Heishankou population, consistent with the gene’s reported role in xanthophore production and previous findings that it is upregulated in yellow vs. black fire salamanders. Jin, Aguilar Gómez, and colleagues identified five divergent paralogs of *slc2a11* in the *P. versicolor* genome, all of which appeared to be under selection and four of which were differentially expressed. The authors hypothesize that the reduced expression of *slc2a11* in the Heishankou population may explain the lower proportion of xanthophores observed in melanic *P. versicolor* lizards.

Interestingly, the authors also found evidence for balancing selection acting on the *slc2a11* locus in the Guazhou County population. According to the study authors, there are two potential explanations for this finding. As this group of lizards is geographically close to the black Heishankou population, members of this population may occasionally find themselves in dark sand habitats, where their yellow phenotype is presumably disfavored. Alternatively, the Guazhou County population may receive divergent haplotypes via gene flow from the neighboring Heishankou population. As the authors point out, “Both scenarios involve balancing selection acting through spatially varying selective pressures for the yellow-associated alleles.”

Both Aguilar Gómez and Jin acknowledge that additional studies are needed to confirm their findings, pointing out the small sample sizes for their microscopic analyses and the low coverage used for resequencing. According to Jin, “The most difficult question is how we can functionally illustrate the role of different alleles on color phenotypes. Technically, it is challenging because the generation time of these lizards is 1–2 years.” Despite these limitations, Aguilar Gómez notes that their study has produced “one of the very few genomic resources for agamid lizards,” with only one other agamid genome (*Pogona vitticeps*) being publicly available. The authors hope that the *P. versicolor* genomes and transcriptomes from this study will be a valuable resource, driving additional investigations into the evolution and natural history of this lineage.
